# Spontaneous Recurrent Hemarthrosis of the Knee: A Report of Two Cases with a Source of Bleeding Detected during Arthroscopic Surgery of the Knee Joint

**DOI:** 10.1155/2016/1026861

**Published:** 2016-09-14

**Authors:** Eisuke Nomura, Hisatada Hiraoka, Hiroya Sakai

**Affiliations:** Department of Orthopaedic Surgery, Saitama Medical Center, Saitama Medical University, Kawagoe, Saitama, Japan

## Abstract

We report two cases of the spontaneous recurrent hemarthrosis of the knee. In these cases lateral meniscus was severely torn and a small tubular soft tissue with pulsation was identified on the synovium in the posterolateral corner during arthroscopic surgery of the knee joint. Gentle grasping of this tissue by forceps led to pulsating bleeding, which stopped by electrocoagulation. This soft tissue was considered a source of bleeding, since no recurrence of hemarthrosis was observed for more than four years after surgery. It was highly probable that this soft tissue was the ruptured end of the lateral inferior genicular artery or its branch. This case report strongly supports the theory that the bleeding from the peripheral arteries of the posterior portion of the lateral meniscus is the cause of spontaneous recurrent hemarthrosis of the knee.

## 1. Introduction

Spontaneous recurrent hemarthrosis of the knee joint is relatively rare disorder mostly seen in the elderly with osteoarthritis. Since the first report of Wilson [1], synovium had been considered the origin of the bleeding [2, 3], and synovectomy was considered to be the most reasonable treatment. In 1994, however, Kawamura et al. [4] reported five cases of spontaneous recurrent hemarthrosis of the knee in which a degenerative flap tear of the posterior horn of lateral meniscus was revealed with arthroscopic examination. These patients did not experience the recurrent hemarthrosis after arthroscopic resection of the injured lateral meniscus, suggesting that the origin of the bleeding was the peripheral arteries of the posterior horn of the lateral meniscus [4]. Since this report, there have been some reports supporting this theory [5–8]. In some cases, bleeding from the posterior portion of the lateral meniscus was observed during arthroscopic examination [4, 6, 7]. Among these reports, Sasho et al. [7] detected throbbing bleeding indicating arterial bleeding in the process of debridement of lateral meniscus, which suggested direct bleeding from the lateral genicular artery. However, the arterial structure itself as a bleeding source was not detected in any case. We report two cases of the spontaneous recurrent hemarthrosis of the knee in which a pulsating soft tissue with a tubular structure as a source of bleeding was identified in the posterolateral corner during arthroscopic surgery of the knee joint.

## 2. Case Report

### 2.1. Case 1

**Figure 1:**
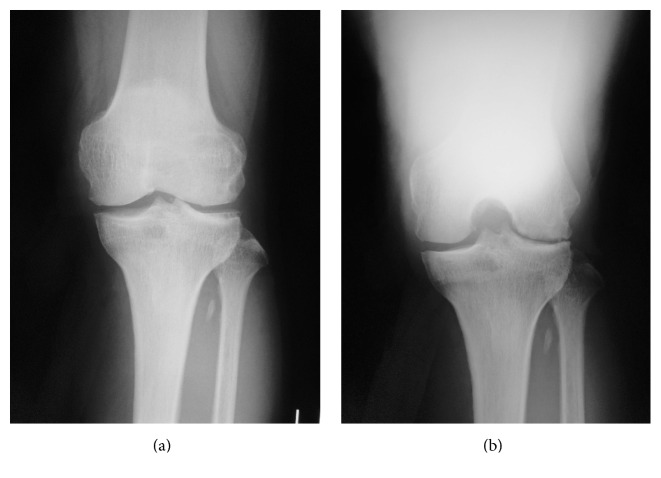
Radiographs of Case 1. AP view (a) shows grade 3 osteoarthritis according to Kellgren and Lawrence scale and Rosenberg view (b) shows lateral-dominant osteoarthritis.

**Figure 2:**
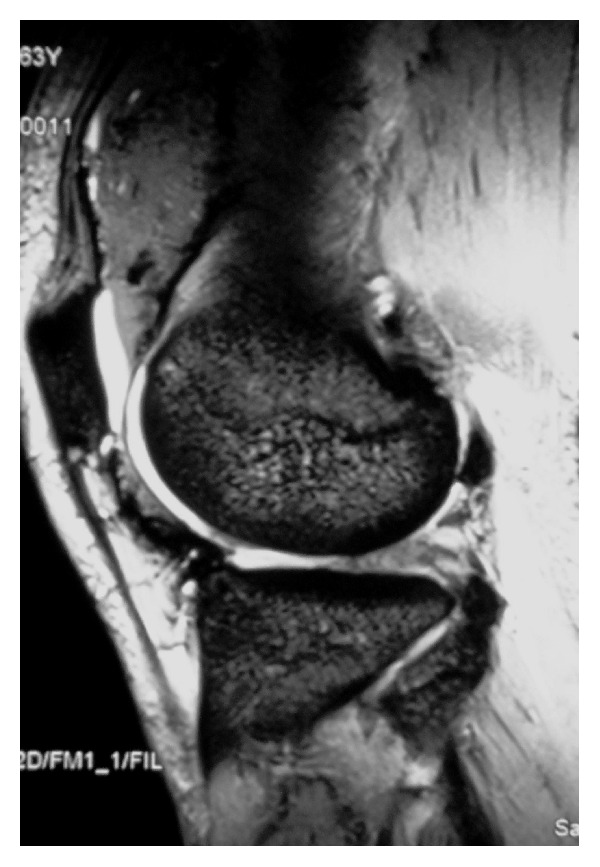
MRI of Case 1 (T2 weighted image). The posterior portion of the lateral meniscus is torn.

A 64-year-old man presented with recurrent painful swelling of the left knee for one month without any traumatic episode. His medical history was unremarkable and only some anti-inflammatory drugs were taken sporadically for the knee pain after hemarthrosis occurred. He received knee joint puncture three times in one month and bloody fluid was aspirated each time. Radiographs of his left knee showed lateral-dominant osteoarthritis (Figure 1), and MRI showed that the posterior portion of the lateral meniscus was torn (Figure 2).

**Figure 3:**
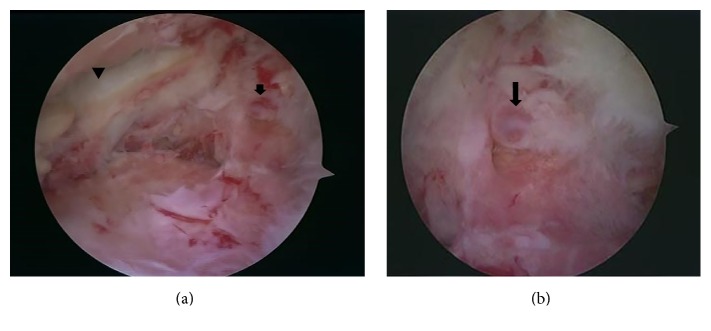
Arthroscopic findings of Case 1. The lateral compartment has severe degenerative change and almost no meniscal substance, including the meniscal rim, is observed in the middle and posterior portions of the lateral meniscus. Arrow head indicates the remaining posterior horn of the lateral meniscus (a). On the synovium behind this area, a small projecting tubular soft tissue with pulsation (arrows) is shown (a and b).

At four months after the onset of the symptom, surgery was performed without using tourniquet. Arthroscopic examination revealed that the lateral compartment had severe degenerative change and that almost no meniscal substance, including the meniscal rim, was observed in the middle and posterior portions of the lateral meniscus, although the posterior horn remained. On the exposed synovium behind this area, a 3-4 mm wide projecting tubular soft tissue was identified (Figure 3). It was pulsating, and when gently grasping this tissue with forceps, pulsating bleeding from the top of this soft tissue was shown, which stopped by electrocoagulation. No recurrence of hemarthrosis was observed at 54 months postoperatively.

### 2.2. Case 2

**Figure 4:**
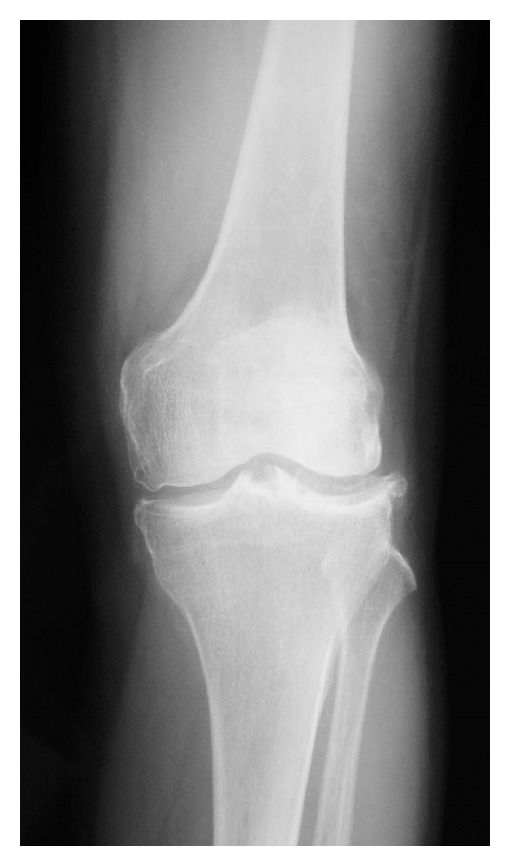
Radiograph of Case 2. AP view shows grade 3 lateral-dominant osteoarthritis according to Kellgren and Lawrence scale.

**Figure 5:**
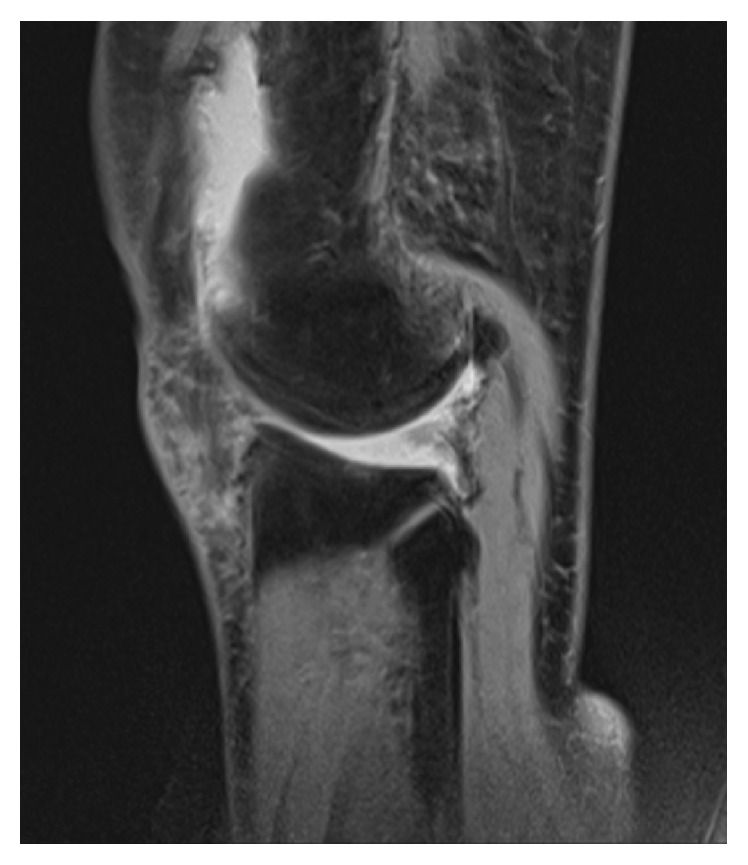
MRI of Case 2 (T2 weighted image). The middle and posterior portions of the lateral meniscus are torn.

A 71-year-old woman presented with two-year history of recurrent swelling of the left knee. The swelling always occurred after sport activity, but she did not have clear history of trauma. She was hypertensive and took hypotensors, but her medical history was otherwise unremarkable. Within those two years, she had her left knee punctured four times with bloody fluid aspiration. Radiographs of her left knee showed lateral-dominant osteoarthritis (Figure 4), and MRI showed that the middle and posterior portions of the lateral meniscus were torn (Figure 5).

**Figure 6:**
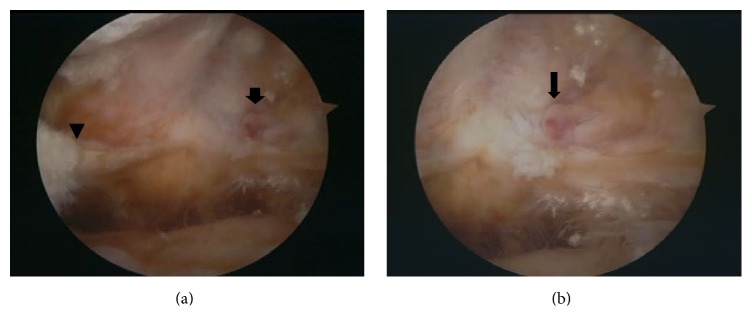
Arthroscopic findings of Case 2. Remarkable degenerative change is shown in the lateral compartment. The middle and posterior portions of the lateral meniscus are degeneratively torn (arrow head) and almost no meniscal substance was left in these portions (a). On the exposed synovium behind this area, a small projecting tubular soft tissue with pulsation (arrows) is shown (a and b).

At 28 months after the onset of the symptom, surgery was performed without tourniquet. Arthroscopic examination demonstrated that the lateral compartment had remarkable degenerative change. And the middle and posterior portions of the lateral meniscus were degeneratively torn and almost no meniscal substance was left in these portions. On the exposed synovium behind this area, a 2-3 mm long projecting tubular soft tissue with a 3-4 mm diameter was identified (Figure 6). It was pulsating and on its top a red spot, suggesting coagulated blood clot, was observed. Gentle grasping with forceps led to pulsating bleeding from the top of this soft tissue, which ceased by electrocoagulation. There was no recurrence of hemarthrosis for 64 months after surgery.

## 3. Discussion 

Regarding the etiology of the spontaneous recurrent hemarthrosis of the knee joint in elderly, synovium had been considered the origin of the bleeding until the report of Kawamura et al. [1–4]. Since the report of Kawamura et al. [4], the peripheral arteries of the posterior portion of the lateral meniscus have been recognized as the origin of the bleeding in most cases [5–8]. In some reports, the bleeding from the posterior portion of the lateral meniscus was observed during arthroscopic surgery [4, 6, 7]. This finding, together with the fact that the recurrence of the hemarthrosis did not occur after lateral meniscectomy alone [4–6, 8], or lateral meniscectomy followed by coagulation [7], was the ground of the theory that the origin of the bleeding was the peripheral arteries of the posterior portion of the lateral meniscus. In these reports, however, the arterial structure itself at the posterior portion of lateral meniscus, which would be the direct evidence of this theory, was not detected during surgery.

We reported two cases of the spontaneous recurrent hemarthrosis of the knee, in which a pulsating soft tissue with a tubular structure was identified on the exposed synovium in the posterolateral corner during arthroscopic surgery. Taking account of its shape, the existing of pulsation, and pulsating bleeding from the top by gentle grasping, it was highly probable that this soft tissue was a ruptured end of the artery. Electrocoagulation of this soft tissue led to no recurrence of hemarthrosis after surgery for more than four years, indicating that this was the source of bleeding in our cases. From the anatomical standpoint, it was the ruptured end of the lateral inferior genicular artery or its branch. Arnoczky and Warren [9] demonstrated that lateral inferior genicular artery was located very close to the peripheral border of the lateral meniscus. Sasho et al. [7] suggested that the throbbing bleeding during lateral meniscectomy was due to direct bleeding from lateral genicular artery.

The lateral inferior genicular artery and its branches supply the posterior portion of the lateral meniscus [9]. In our cases, the posterior portion of the lateral meniscus was severely torn and almost no meniscal substance, including the meniscal rim, was observed, suggesting that when the lateral meniscus was torn from its junction with the synovium, the artery was ruptured with its end left on the exposed synovium and was identified as a pulsating soft tissue during surgery. Therefore, our cases strongly support the theory that the bleeding from the peripheral arteries of the posterior portion of the lateral meniscus is the cause of spontaneous recurrent hemarthrosis of the knee. Although Sasho et al. [7] observed throbbing bleeding indicating arterial bleeding during lateral meniscectomy, the arterial structure itself was not detected. As far as we know, there was only one case report showing the ruptured end of the pulsating vessel in the patient of the recurrent hemarthrosis of the knee [10]. In this report, the ruptured end of pulsating vessel behind the rim of lateral meniscus was observed during arthroscopic examination and the coagulation of the vessel led to no recurrence of the hemarthrosis [10]. The operative findings of this case were very similar to those of our cases.

Regarding the cause of the bleeding from the artery in spontaneous recurrent hemarthrosis, Kawamura et al. [4] suggested that the pulling and laceration into the branches of the genicular arteries supplying the peripheral rim of the lateral meniscus might be the direct cause of the hemorrhage into the joint. In our cases, however, the pulling of the artery was not likely to occur, because almost no meniscus substance was left, causing no continuity of the artery with the meniscus. In our cases, it was most likely that the thrombus created at the end of artery was detached due to mechanical irritation with knee motion, causing bleeding into the joint, that the increased intraarticular pressure caused by bleeding led to the stop of the bleeding with thrombus formation at the end of the artery, and that this thrombus was detached later, causing intraarticular bleeding again. This cycle was likely the cause of the recurrent hemarthrosis in our cases. In our two cases, it was likely that grasping the end of the artery during surgery caused the detachment of the thrombus, leading to the bleeding, and that the electrocoagulation was successful in permanent closing of the end of the artery.

Although medial-dominant osteoarthritis is more common than lateral-dominant osteoarthritis, most of the cases with spontaneous recurrent hemarthrosis of the knee are associated with lateral-dominant osteoarthritis and/or lateral meniscal injury [4–8]. This may be explained by the location and size of the genicular arteries. The lateral inferior genicular artery courses adjacent to the peripheral border of the lateral meniscus [9], whereas the medial genicular arteries do not course so close to the medial meniscus. Also the lateral inferior genicular artery is much larger than the medial genicular arteries [11, 12].

We did not use tourniquet from the beginning of the surgery, which enabled us to detect the pulsation of the soft tissue. If we had used the tourniquet, this might not have been detected in our cases. When a patient of recurrent hemarthrosis of the knee is encountered, especially when such a patient has lateral-dominant osteoarthritis and/or torn lateral meniscus, it is highly probable that the origin of the bleeding is the branch of lateral inferior genicular artery that penetrates and supplies the lateral meniscus. And in the beginning of the arthroscopic surgery for such a case, the use of the tourniquet is not recommended.
